# The cyclin G-associated kinase (GAK) inhibitor SGC-GAK-1 inhibits neurite outgrowth and synapse formation

**DOI:** 10.1186/s13041-022-00951-6

**Published:** 2022-07-26

**Authors:** Jun Egawa, Reza K. Arta, Vance P. Lemmon, Melissa Muños-Barrero, Yan Shi, Michihiro Igarashi, Toshiyuki Someya

**Affiliations:** 1 Department of Psychiatry, School of Medicine, and Graduate School of Medical and Dental Sciences, Niigata University, 757 Asahimachi Dori-Ichibancho, Chuo-ku, Niigata, 951-8510 Japan; 2 Department of Neurochemistry and Molecular Cell Biology, School of Medicine, and Graduate School of Medical and Dental Sciences, Niigata University, 757 Asahimachi Dori-Ichibancho, Chuo-ku, Niigata, 951-8510 Japan; 3 Miami Project to Cure Paralysis, University of Miami Miller School of Medicine, Miami, FL USA; 4 Institute for Data Science and Computing, University of Miami Miller School of Medicine, Miami, FL USA

**Keywords:** Cyclin G-associated kinase (GAK), SGC-GAK-1, Primary neuron culture, High content screening

## Abstract

Protein kinases are responsible for protein phosphorylation and are involved in important signal transduction pathways; however, a considerable number of poorly characterized kinases may be involved in neuronal development. Here, we considered cyclin G-associated kinase (GAK) as a candidate regulator of neurite outgrowth and synaptogenesis by examining the effects of the selective GAK inhibitor SGC-GAK-1. SGC-GAK-1 treatment of cultured neurons reduced neurite length and decreased synapse number and phosphorylation of neurofilament 200-kDa subunits relative to the control. In addition, the related kinase inhibitor erlotinib, which has distinct specificity and potency from SGC-GAK-1, had no effect on neurite growth, unlike SGC-GAK-1. These results suggest that GAK may be physiologically involved in normal neuronal development, and that decreased GAK function and the resultant impaired neurite outgrowth and synaptogenesis may be related to neurodevelopmental disorders.

**Supplementary Information:**

The online version contains supplementary material available at 10.1186/s13041-022-00951-6.

Phosphorylation is considered to be the most important protein modification in signal transduction pathways. Protein kinases are the responsible enzymes for this process, and there are more than 500 species of kinases encoded in the human genome [1]. However, most of these are not well characterized, and their exact biological roles or functions are not known. Neurons have more complex signaling pathways than other cells because of their role in synaptic plasticity [2], and there are potentially numerous events that involve uncharacterized protein kinases. For example, c-jun *N*-terminus protein kinase (JNK) is reported to be tightly related to axon growth and regeneration [3].

Cyclin G-associated kinase (GAK) is one such kinase that is not well characterized. This protein is a 160 kDa serine/threonine kinase, and the structure is comprised of a kinase domain at the *N*-terminus, a PTEN-like domain, a clathrin binding domain, and a C-terminal J domain [4, 5]. Neuron-specific GAK knockouts cause defects in the proliferation of neural progenitor cells in the hippocampal region, causing neuronal depletion and changes in brain morphology, and mouse pups were observed to survive only a few days after birth [6]. However, the effects of GAK on neurodevelopmental processes have not been well investigated. Therefore, we used SGC-GAK-1, which had an IC_50_ of 48 nM in the live cell target engagement assay [7, 8].

The experimental procedures are described in detail in Additional file 1. We investigated how selective inhibition of GAK using SGC-GAK-1 [9–12] affects neurite outgrowth and synaptogenesis in mouse hippocampal neurons, according to the scheme shown in Fig. 1A. For quantification, a high-throughput screening system, the microscope-based CellInsight™ CX5 High Content Screening platform (Thermo Fisher Scientific), was used. First, we compared the total neurite length per number of neurons of samples treated with eight concentrations of SGC-GAK-1 compared to the control as above. Total neurite length per number of neurons was significantly reduced at 5, 10, 20, and 40 μM SGC-GAK-1 compared to the control supplemented with DMSO alone (*p* < 0.05/8; Fig. 1B, C). Total neurite branch points per number of neurons were also significantly reduced at 10, 20, and 40 μM SGC-GAK-1 compared to the control supplemented with DMSO alone (*p* < 0.05/8; Additional file 2: Fig. S1). There was no significant difference in the number of neurons from 0.3125 to 10 μM SGC-GAK-1 compared to the control (Additional file 1: Table S1). In addition, we measured synapse formation using double-immunostaining of synaptophysin (presynaptic marker) and SHANK2 (postsynaptic marker), and the total number of synapses per number of neurons was also significantly reduced at 10, 20, and 40 μM SGC-GAK-1 compared to the control (DMSO alone; *p* < 0.05/8; Fig. 1D–F). There was also no significant difference in the number of neurons from 0.3125 to 5.0 μM SGC-GAK-1 compared to the control (Additional file 1: Table S1).

**Fig. 1:**
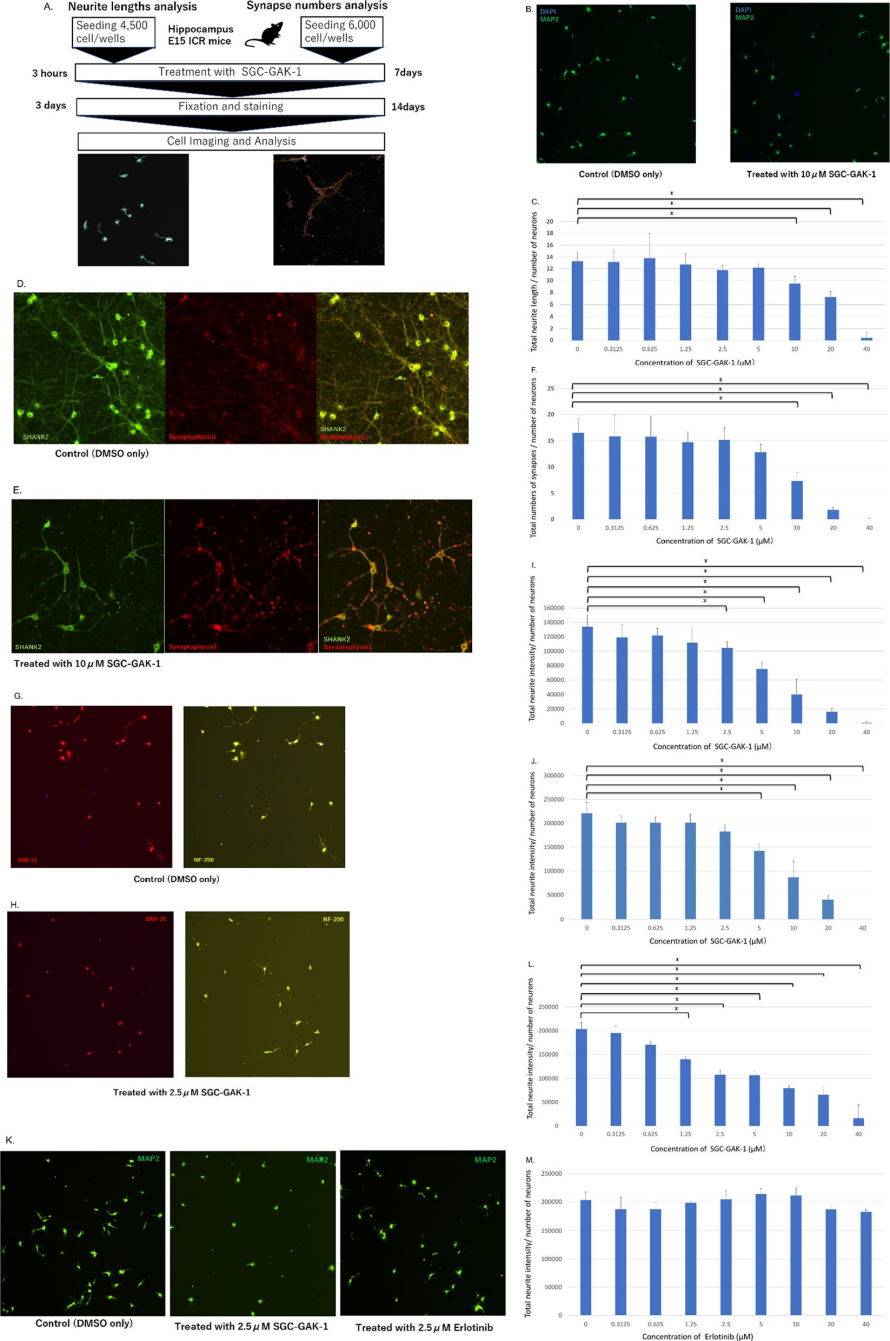
Effects of GAK inhibition on neurodevelopment in mice. **A** Outline of the procedure. The ROI is shown within the yellow dotted curves, in which synaptic puncta were quantified (*Right*). **B** Cell imaging of neurons treated with 10 μM SGC-GAK-1 or dimethysulfoxide (DMSO) as the control. **C** Total neurite length per number of neurons in samples treated with 0.3125, 0.625, 1.25, 2.5, 5, 10, 20, and 40 μM SGC-GAK-1 or DMSO (N = 6, each concentration). **D** Cell imaging of neurons treated with DMSO and stained with anti-synaptophysin 1 and anti-SHANK2 antibodies. **E** Cell imaging of neurons treated with 10 μM SGC-GAK-1 and stained with anti-synaptophysin 1 and anti-SHANK2 antibodies. **F** Total number of synapses per number of neurons in samples treated with SGC-GAK-1 (same concentrations as in **C**) or DMSO (N = 6, each concentration). **G** Cell imaging of neurons treated with DMSO and stained with SMI-31 or NF-200. **H** Cell imaging of neurons treated with 2.5 μM SGC-GAK-1 and stained with SMI-31 or NF-200. **I** SMI-31 staining: Neurite intensity per number of neurons in samples treated with SGC-GAK-1 (same concentrations as in **C**) or DMSO. **J** NF200 staining: Neurite intensity per number of neurons in samples treated with SGC-GAK-1 (same concentrations as in **C**) or DMSO (N = 3, each concentration). **K** Cell imaging of neurons treated with 2.5 μM SGC-GAK-1, 2.5 μM erlotinib, or DMSO and stained with MAP2. **L** SGC-GAK-1 treatment: Neurite intensity per number of neurons (stained with MAP2) in samples treated with SGC-GAK-1 (same concentrations as in **C** and N = 3, each concentration) or DMSO (N = 6). **M** Erlotinib treatment: Neurite intensity per number of neurons (stained with MAP2) in samples treated erlotinib (N = 3, each concentration, which is the same as in **C**) or DMSO (N = 6). Image size: 899.04 µm × 899.04 µm (**B**, **G**, **H**, **K**); 455.44 µm × 455.44 µm (**D**, **E**). Total neurite length/number of neurons, total number of synapses/number of neurons, and total neurite intensity/number of neurons were significant (*) at p < 0.05/8 after Bonferroni’s correction and the error bars indicate standard deviation

Next, we immunostained the cultured neurons after exposure to SGC-GAK-1, using NF-200 and SMI-31 antibodies specific to the phosphorylated neurofilament (NF)-200 kDa subunit, and analyzed the immunoreactivity intensity in neurites. In both SMI-31 and NF-200 staining, the neurite intensity decreased in proportion to the increase in SGC-GAK-1 concentration (*p* < 0.05/8). However, at 2.5 μM, a significant difference was observed only in the intensity of SMI-31 compared to the control (Fig. 1G–J). The result showed that SGC-GAK-1 inhibits the phosphorylation function of GAK even in the concentration range with low neuronal cytotoxicity. Thus, we concluded that phosphorylation of the NF-200 kDa subunit was inhibited by SGC-GAK-1 in growing neurites.

We compared the neurite intensity of neurons treated with SGC-GAK-1 and erlotinib, another protein kinase inhibitor structurally related to SGC-GAK-1 but with lower specificity to GAK [10, 13]. Neurons were treated with erlotinib as described for SGC-GAK-1. In neurons treated with 1.25, 2.5, 5, 10, and 20 μM SGC-GAK-1, the neurite intensity significantly decreased compared to the control (*p* < 0.05/8). Among them, there were no significant differences in the number of neurons for the 1.25, 2.5, and 5 μM concentrations (Fig. 1K–M). The results showed that SGC-GAK-1 more specifically affected neuronal development involving GAK than did erlotinib. SGC-GAK-1 is a specific inhibitor of GAK, making it an ideal choice for assessing GAK activity. The narrow kinome spectrum and potent cell target engagement make it a better choice for deconvoluting GAK biology than a clinical kinase inhibitor such as erlotinib, which primarily targets EGF receptor tyrosine kinases and has off-target effects on GAK [10, 13]. Erlotinib did not show effects similar to those of SGC-GAK-1 (Fig. 1K–M), indicating that the latter inhibitor affected neurite outgrowth and synaptogenesis via GAK specifically.

Neurodevelopmental disorders, such as autism spectrum disorders, which affect communication, cognition, social interaction, and other patterned behaviors, are currently known to be caused partly by genetic mutations of brain signaling molecules [14] including protein kinases [15]. As shown here, GAK is involved in the normal development of neurons, suggesting the possibility that this or related kinases play a role in the pathogenesis of such diseases. Previously, GAK was reported to be related to neuronal degeneration in Parkinson’s disease by its enhancement of α-synuclein-mediated toxicity [16, 17]; however, no evidence for its relationship to neuronal development has been demonstrated to date. Clathrin adapter proteins were reported to be potential substrates for this kinase in in vitro experiments [18], and an ongoing search for other physiological and specific substrates for this kinase is needed.

In conclusion, using the high-throughput imaging quantification system, we showed that axon outgrowth and synaptogenesis are impeded by inhibiting the phosphorylation function of GAK. It was also demonstrated that SGC-GAK-1 inhibits the substrate phosphorylation function of GAK, and affects neurodevelopment to a greater extent than erlotinib. The novel findings of the present study demonstrated that reduced GAK function is associated with neurodevelopmental disorders as well as α-synuclein-mediated neurodegeneration.

## Supplementary Information

**Additional file 1: Table S1.** The number of neurons

**Additional file 2: Figure S1.** Neurite branches were inhibited by SGC-GAK-1.

## Data Availability

All data generated or analyzed during this study are included in this published article.
