# Supplementary figures and images for: The cyclin G-associated kinase (GAK) inhibitor SGC-GAK-1 inhibits neurite outgrowth and synapse formation
Source: Mol Brain. 2022 Jul 26;15:68. doi: 10.1186/s13041-022-00951-6 (PMC9327206; doi:10.1186/s13041-022-00951-6)

**
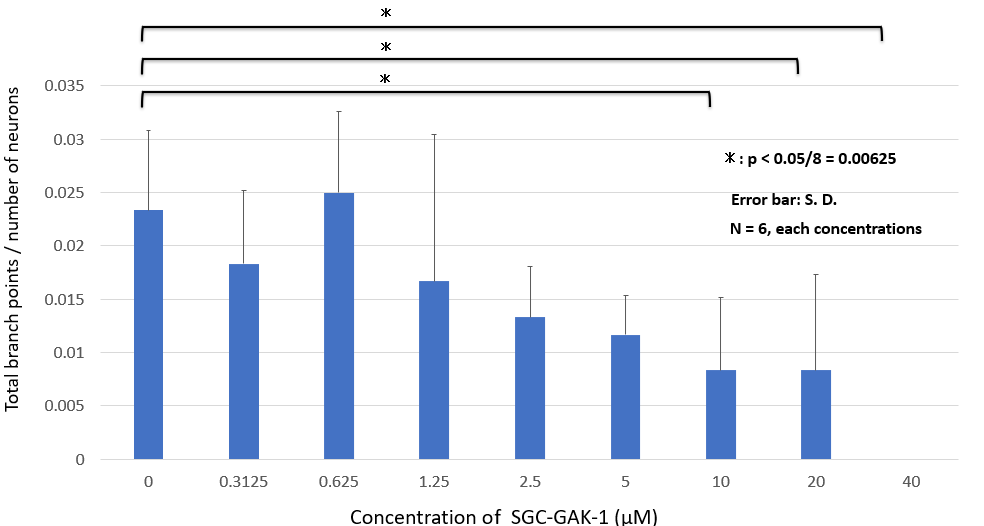
Supplementary Figure S1: Analysis of neurite branch points of SGC-GAK-1 treated neurons**

Supplement: Supplementary file 1 — Additional file 1: Table S1. The number of neurons [file 13041_2022_951_MOESM1_ESM.docx]
