# Supplementary material for: The cyclin G-associated kinase (GAK) inhibitor SGC-GAK-1 inhibits neurite outgrowth and synapse formation
Source: Mol Brain. 2022 Jul 26;15:68. doi: 10.1186/s13041-022-00951-6 (PMC9327206; doi:10.1186/s13041-022-00951-6)
Supplement: Supplementary file 2 — Additional file 2: Figure S1. Neurite branches were inhibited by SGC-GAK-1. [file 13041_2022_951_MOESM2_ESM.docx]

**Supplemental Information**

**Supplemental Methods**

***Animals***

All animal experiments were performed following approval from the Animal Resource Center of Niigata University. Pregnant ICR mice were purchased from Japan-SLC, Inc. (Shizuoka, Japan).

***Primary Cell Dissociation and Cell Plating***

Hippocampal neurons of E15 mice were cultured as described previously [19]. Sixty wells of 96-well plates were filled with 200 µL of neuronal culture medium consisting of Neurobasal™ Plus Medium (Thermo Fisher Scientific) with 2% B-27. Cells were resuspended at a concentration of 4,500 cells/100 µL for neurite length analysis or 6000 cells/100 µL for synapse number analysis, filtered, then dispensed into plates at a final volume of 200 µL.

***Treatment with Kinase Inhibitors***

SGC-GAK-1 and erlotinib were purchased from Bio-Techne/TOCRIS Japan. For neurite length analysis, plates were incubated for 3 hours in 95% humidity at 37°C prior to the addition of SGC-GAK-1 (in 0.8% DMSO) to each well of the culture plate at final concentrations of 0.3125, 0.625, 1.25, 2.5, 5.0, 10, 20, and 40 µM; 0.8% DMSO was added to the control wells. The plate was then incubated for 3 days under the same conditions. For synapse numbers analysis, plates were incubated for 7 days in 95% humidity at 37°C prior to the addition of SGC-GAK-1 (in 0.8% DMSO) to each well of the culture plate at final concentrations of 0.3125, 0.625, 1.25, 2.5, 5.0, 10, 20, and 40 µM; 0.8% DMSO was added to the control wells. The plate was then incubated for 14 days under the same conditions.

Erlotinib treatment was performed after plates were incubated for 3 hours in 95% humidity at 37°C prior to the addition of erlotinib (in 0.8% DMSO) to each well of the culture plate at final concentrations of 0.3125, 0.625, 1.25, 2.5, 5.0, 10, 20, and 40 µM; 0.8% DMSO was added to the control wells. The plate was then incubated for 3 days under the same conditions.

***Fixation and Staining***

Neurons were fixed at room temperature for 15 min with 100 µL of 4% paraformaldehyde in phosphate-buffered saline (PBS). The cells were washed three times with PBS and then blocked overnight at room temperature with 5% bovine serum albumin and 0.03% Triton-X100 in PBS. Primary antibody staining was performed overnight at 4°C in the same buffer containing antibodies that recognized MAP2 (1:300, Cat# PA5-85755, rabbit, Invitrogen) only for neurite length analysis, or synaptophysin 1 (1:400, Cat# 101 011, mouse, Synaptic Systems), SHANK2 (1:400, Cat# 162204, guinea pig, Synaptic Systems), and MAP2 (1:300, Cat# PA5-85755, rabbit, Invitrogen) for synapse number analysis. SMI-31 (1:1000, Cat# NE1022, mouse, Sigma-Aldrich) and NF200 (1:500, Cat# N4142, rabbit, Sigma-Aldrich) were used to confirm the inhibition of GAK phosphorylation. The next day, the cells were washed three times with PBS, incubated for 2 hours at room temperature with fluorescently labeled secondary antibodies (1:500, Cat# 706-545-148, donkey anti-guinea pig-IgG (H+L)-Alexa488, Jackson IR; 1:500, Cat# A10042, donkey anti-rabbit-IgG (H+L)-Alexa568, Invitrogen; 1:500, Cat# A31571, donkey anti-mouse-IgG (H+L)-Alexa647, Jackson) and 1:1000 DAPI. The cells were then washed five times with PBS. All washing steps were performed with a robotically operated Wellwash Versa (Thermo Fisher Scientific). Brightfield, nuclei, and GFP images of the cells were then acquired with the microscope-based CellInsight^TM^ CX5 High Content Screening (HCS) platform (Thermo Fisher Scientific) using a 10X (for neurite length analysis) or 20X (for synapse number analysis) objective lens.

***Image Acquisition and Analysis***

Image acquisition was performed using a CellInsight^TM^ CX5. This instrument analyses epifluorescence of individual cell events using an automated microplate reader interfaced with a PC (Dell precision 136 T5600 workstation). DAPI staining was used to identify nuclei. MAP2 with Alexa568 was used as a neurite marker, and synaptophysin 1 with Alexa647 was used as a synaptic marker. Neurons were identified based on MAP2 and DAPI signals, and their numbers were quantified. In the neurite length analysis, neurites were detected by the MAP2 signal. The total of calculated neurite lengths and neurite branch points in the entire visual field divided by the number of neurons [20] was compared. In the synapse number analysis, a synapse signal was defined as the synaptophysin 1 signal being ≥ 50% proximal to a MAP2 neurite signal. Putative positive signals and control images were also analyzed using a custom algorithm developed using the Neuronal Profiling BioApplication v4.2 (ThermoFisher). All data are expressed as mean ± the standard error of the mean. All statistical analyses were conducted using BellCurve for Excel software. Control cells (treated with 0.8% DMSO) and cells with each kinase inhibitor concentration (in 0.8% DMSO) were compared using Student's t-test or Welch's t-test. An alpha level of *p* < 0.05 was considered statistically significant, and multiple comparisons were corrected using Bonferroni’s method.

**Supplemental References**

19. Nozumi M, Nakatsu F, Katoh K, Igarashi M. Coordinated Movement of Vesicles and Actin Bundles during Nerve Growth Revealed by Superresolution Microscopy. Cell Rep. 2017; 18: 2203-2216.

20. Buchser WJ, Slepak TI, Gutierrez-Arenas O, Bixby JL, Lemmon VP. Kinase/phosphatase overexpression reveals pathways regulating hippocampal neuron morphology. Mol Syst Biol. 2010; 6: 391.

**Legends to the Supplementary Figure**

**Supplementary Fig. S1. Neurite branches were inhibited by SGC-GAK-1.** Total neurite branch points per number of neurons were also significantly reduced at 10, 20, and 40 μM SGC-GAK-1 compared to the control supplemented with DMSO alone (*p* < 0.05/8).

**Supplementary Table 1**

**The number of neurons**

|  | **Concentration of SGC-GAK-1 (μM)** | **0**  **(Control)** | **0.3125** | **0.625** | **1.25** | **2.5** | **5.0** | **10** | **20** | **40** |
| --- | --- | --- | --- | --- | --- | --- | --- | --- | --- | --- |
| **Culture period** |  |  |  |  |  |  |  |  |  |  |
| 3 days |  | 160.5 ± 13.35 | 174.5 ± 11.38 | 207.7 ± 22.36 | 227.3 ± 18.82 | 204.3 ± 43.17 | 192.7 ± 41.97 | 161.2 ± 32.11 | 97.2 ± 20.06* | 0.8 ± 1.33* |
| 14 days |  | 313.5 ± 143.35 | 314.2 ± 82.44 | 272.5 ± 57.18 | 278.0 ± 108.62 | 313.3 ± 123.63 | 248.5 ± 90.26 | 132.8 ± 26.57* | 40.8 ± 8.59* | 0.2 ± 0.41* |

*: Concentration in which the number of cells was significantly reduced, compared to Control (***p*** < 0.05/8)
